# Recombinant expression and functional characterization of influenza A virus neuraminidase in a mammalian cell system

**DOI:** 10.3389/fmicb.2025.1729393

**Published:** 2025-11-12

**Authors:** Ailing Huang, Yulong Liu, Yanbai Li, Zhe Yin, Shanshan Huo, Juan Wang, Zhenlin Yang, Tianlei Ying, Fei Yu

**Affiliations:** 1 Hebei Key Laboratory of Analysis and Control of Zoonotic Pathogenic Microorganism, College of Life Sciences, Hebei Agricultural University, Hebei, China; 2 Key Laboratory of Medical Molecular Virology (MOE/NHC/CAMS), Shanghai Institute of Infectious Disease and Biosecurity, School of Basic Medical Sciences, Fudan University, Shanghai, China; 3 Shanghai Engineering Research Center for Synthetic Immunology, Shanghai, China; 4 Shanghai Key Laboratory of Lung Inflammation and Injury, Department of Pulmonary Medicine, Zhongshan Hospital, Fudan University, Shanghai, China

**Keywords:** influenza A virus, neuraminidase, tetrameric assembly, mammalian expression, broad-spectrum vaccine

## Abstract

The soluble and functional expression of influenza A virus neuraminidase (NA) remains a major challenge due to its membrane-associated nature and structural complexity. In this study, we established a mammalian expression strategy that enables the production of correctly folded, enzymatically active NA proteins across multiple influenza A subtypes. Through systematic N-terminal deletion mapping, we identified a conserved structural boundary within the transmembrane-stalk region that critically determines NA solubility. Truncation of approximately 65–80 amino acids, combined with enforced tetramerization *via* a human VASP domain, allowed efficient secretion of stable tetrameric NAs in Expi293 cells. The resulting proteins displayed native-like mushroom-shaped symmetry under electron microscopy, exhibited robust enzymatic activity, and retained high binding affinity to the broadly protective antibody 1G01. Immunization with tetrameric NAs elicited strong humoral responses targeting conserved epitopes in the enzymatic active center in mice, indicating that tetrameric NA vaccines efficiently induce protective antibodies. These findings define the structural determinants required for soluble and immunogenic NA expression, and provide a versatile platform for the development of broad-spectrum influenza vaccines and antiviral agents.

## 1 Introduction

Influenza A virus (IAV) causes periodic epidemics that threaten human and animal health worldwide. Despite the availability of vaccines and antiviral agents, the extensive genetic and antigenic variability of IAV continues to challenge the effectiveness of current preventive and therapeutic strategies (Jones et al., 2023). Antigenic drift and occasional antigenic shift alter viral surface glycoproteins and enable the virus to evade host immune surveillance (Wille and Holmes, 2020).

The surface of IAV contains two major glycoproteins, hemagglutinin (HA), and neuraminidase (NA), both of which are essential for the viral life cycle. HA, a trimeric membrane glycoprotein with high antigenicity, mediates viral attachment and membrane fusion by binding to sialic acid-containing receptors on host cells, thereby initiating viral entry (Garcia et al., 2023; Hu et al., 2023). In contrast, NA is a tetrameric glycoprotein that catalyzes the cleavage of terminal sialic acid residues from host and viral glycoconjugates, facilitating the release of progeny virions and preventing their aggregation at the cell surface (Krammer and Palese, 2019). Interference with either protein disrupts viral replication, underscoring their complementary roles in infection and transmission.

With the extensive use of HA-based vaccines and neuraminidase inhibitors, IAV continues to evolve through antigenic drift and antigenic shift, enabling immune escape and reducing vaccine efficacy (Hou et al., 2016; Huang et al., 2022; Lei et al., 2023). Increasing evidence highlights NA as a promising and relatively conserved antigenic target that can complement HA in the design of next-generation influenza vaccines (Govorkova et al., 2022). In addition, the conservation of NA also offers potential for developing broad-spectrum anti-influenza drugs (Momont et al., 2023; Russell et al., 2006; Stadlbauer et al., 2019), further driving the demand for NA research (Lv et al., 2025). In these efforts, obtaining NA proteins with optimal biological function is a critical factor for success. However, the production of recombinant NA proteins that retain native conformation and enzymatic activity across subtypes remains technically demanding. Differences in extracellular domain length, uncertainty in defining the transmembrane-stem boundary, and the challenge of achieving both solubility and correct tetrameric assembly in mammalian systems often result in low yield or misfolded proteins (Ellis et al., 2022; Lipnicanova et al., 2022). These difficulties impede the structural and functional characterization of NA and limit its application in vaccine and antiviral development.

In this study, we developed a mammalian expression strategy to achieve soluble and functionally active production of recombinant NA proteins across multiple influenza A subtypes. By systematically mapping N-terminal truncations, we identified a conserved structural boundary within the transmembrane-stalk region that governs NA solubility and folding. Truncation of approximately 65–80 amino acids, combined with enforced tetramerization through fusion of a human vasodilator-stimulated phosphoprotein (VASP) domain, enabled efficient secretion of properly assembled tetrameric NAs in Expi293 cells. The resulting proteins exhibited high enzymatic activity, preserved binding affinity to the broadly protective antibody 1G01, and elicited epitope-focused antibody responses in mice. This approach provides a generalizable framework for producing structurally intact and immunogenic NA antigens, facilitating both mechanistic studies of NA function and the rational design of broad-spectrum influenza vaccines and inhibitors.

## 2 Materials and methods

### 2.1 Cells and plasmids

*Escherichia coli* HB2151 and TOP10 competent cells were used for plasmid construction and propagation. The mammalian expression vectors pSecTag-2A and pCMV6 were employed for recombinant protein expression. Expi293 cells (Thermo Fisher Scientific) were maintained in expression medium under standard culture conditions (37 °C, 8% CO_2_, 120 rpm) and used for transient transfection.

### 2.2 Main reagents

T4 DNA Ligase, SfiI endonuclease, and NotI endonuclease were purchased from New England Biolabs (NEB) in the United States; the homologous recombination kit was acquired from Suzhou Jinan Protein Technology Co., Ltd.; HRP-conjugated peanut agglutinin (PNA-HRP), fetuin, and uranyl acetate were procured from Sigma-Aldrich; PEI Max transfection reagent was obtained from Polysciences, Inc.; ABTS substrate solution was bought from Thermo Fisher Scientific; His tag purification medium Ni Smart Beads 6FF was sourced from Changzhou Tiandi Renhe Biotechnology Co., Ltd.; and the Expi293 cell culture medium Union 293 was purchased from Shanghai Union Biotechnology Co., Ltd.

### 2.3 Construction of NA recombinant protein expression vectors

We constructed recombinant expression vectors for neuraminidase (NA) by first obtaining the NA sequences of the influenza A viruses H5N8/A/Astrakhan/3212/2020, H2N2/A/mallard/MT/Y61, H10N8/A/Jiangxi/IPB13/2013, H7N9/A/Shanghai/4664 T/2013 and H5N1/A/Thailand/Kan353/2004 from GenBank with accession numbers UJS29066.1, AFJ12544.1, AHK10769.1, AGI60295.1 and ABP52012.1, respectively. These sequences were utilized to design recombinant proteins representing the extracellular domains of both monomeric and tetrameric forms of NA, which were designated as H5N8-mono, H5N8-tet, H2N2-mono, and H2N2-tet. Specifically, the tetrameric NA protein constructs incorporated the tetrameric domain of human VASP at the N-terminus to enhance the formation of tetrameric structures, whereas the monomeric constructs lacked this VASP domain (Kuhnel et al., 2004; Stadlbauer et al., 2019). The sequences were optimized for mammalian cell codon usage and chemically synthesized by Shenzhen Hua Da Gene Technology Co., Ltd. Subsequently, the pSecTag-2A vector or pCMV6 vector was subjected to double-enzyme digestion, followed by gel recovery and integration of the target genes into the vector using a homologous recombination kit, facilitating the generation of the desired expression vectors for subsequent studies.

### 2.4 Expression of NA recombinant proteins

On the day before transfection, Expi293 cells were seeded into culture flasks at an initial density of 0.8 × 10^6^ cells/mL in fresh, sterile culture medium. The flasks were subsequently incubated in a controlled environment shaking incubator set to 8% CO_2_, 120 rpm, and a temperature of 37 °C to maintain optimal cell growth conditions. On the day of transfection, cell counts were performed to ascertain that the density had reached approximately 1.5 × 10^6^ cells/mL, with the cell viability confirmed to be above 99%. A transfection complex was formed by combining the PEI transfection reagent with the recombinant plasmid at a mass ratio of 3:1, and the mixture was incubated at ambient temperature for 15 min to facilitate complexation. This transfection complex was carefully added to the Expi293 cells in a dropwise manner while the culture flask was gently agitated to ensure homogeneous distribution, after which the cells were returned to the incubator for continued cultivation. Five days subsequent to transfection, the supernatant from the cell culture was harvested for downstream protein purification processes.

### 2.5 Purification of NA recombinant proteins

The initial step of protein purification entailed the utilization of a Ni Smart Beads 6FF affinity column to selectively bind and enrich the His-tagged NA recombinant proteins. Subsequent to the affinity capture, a Superdex 200 Increase 10/300GL size-exclusion chromatography column was employed to effect further resolution and purification of the proteins. Following elution, the concentration of the purified proteins was ascertained using a Nanodrop spectrophotometer, providing a quantitative assessment of protein yield. To validate the purity and authenticity of the recombinant proteins, Native-PAGE analysis was conducted, which facilitated the visualization and verification of distinct protein bands corresponding to the expected molecular weights of the NA recombinant proteins.

### 2.6 Negative staining electronic microscopy

To facilitate the visualization of NA recombinant proteins by electronic microscopy, a series of protein samples were prepared at four distinct concentrations: 0.1 mg/mL, 0.05 mg/mL, 0.02 mg/mL, and 0.01 mg/mL. Aliquots of 30 μL for each concentration were stored at 4 °C for subsequent use. Carbon-coated copper grids were rendered hydrophilic through a glow discharge treatment, a process essential for enhancing sample adhesion. The protein samples were applied to the grids in a dropwise manner to ensure even distribution.

Uranyl acetate, serving as the negative stain, was then applied to the grids to interact with the proteins and enhance contrast. Following a specified staining duration, the excess stain was meticulously removed, and the grids were air-dried to prevent any sample degradation or contamination. The grids, with the stained protein samples, were examined using a transmission electron microscope. The electron beam provided by the microscope interacted with the samples, yielding high-resolution images that delineated the ultrastructure of the negatively stained NA recombinant proteins, thereby facilitating a detailed morphological analysis.

### 2.7 Mouse immunization

In immunization experiments uisng Balb/c mice, NA recombinant proteins were employed as the immunogen, and aluminum adjuvant was utilized to enhance the immune response. The immunogen preparation involved the combination of 30 μg of NA recombinant proteins with an equal volume of aluminum adjuvant, followed by vigorous mixing using a vortex mixer to ensure a homogeneous suspension. Mice were immunized by administering 100 μL of this emulsion via intramuscular injection, with an equal distribution of 50 μL into each hind leg. The immunization schedule entailed a total of three vaccinations, administered at 14-day intervals, with blood collection performed on the 21st and 35th days post-initial immunization.

Ocular micro-blood collection techniques were applied, specifically at the inner canthus, to collect a precise volume of 150 μL from each mouse. The collected blood samples were transferred into 1.5 mL tubes and incubated at room temperature for 1 h to allow for clot retraction. The tubes were then centrifuged at 6000 rpm for 15 min at 4 °C to separate the serum from the cellular components. Post-centrifugation, the supernatant serum was carefully extracted and aliquoted into fresh tubes for long-term storage at −20 °C, ensuring the preservation of serum samples for subsequent immunological analyses.

The animal experimental protocol involved in this study has been approved by the Ethics Committee of the Basic Medical Sciences of Fudan University (approval number: 20220311–001).

### 2.8 Enzyme-linked lectin assay (ELLA)

The Enzyme-Linked Lectin Assay (ELLA) is a method utilized to evaluate the enzymatic activity of neuraminidase (NA) recombinant proteins by measuring the binding of fetuin, post-cleavage by NA recombinant proteins, to horseradish peroxidase (HRP)-conjugated peanut agglutinin (PNA-HRP) (Lambre et al., 1990). The assay protocol commenced with the addition of 20 μg of fetuin per well in a 96-well microplate, followed by overnight incubation at 4 °C. Subsequent to this, the microplate underwent three washes with PBST to eliminate unbound fetuin. A blocking step ensued with 5% bovine serum albumin (BSA) for 2 h at ambient temperature. In parallel, a fresh 96-well dilution plate was prepared, containing NA recombinant proteins diluted in a 1:2 serial dilution, initiating at a concentration of 20 μg/mL, yielding a total of 24 dilutions, each in duplicate. These diluted NA recombinant proteins were transferred to the wells of the ELISA plate and incubated for 18 h at 37 °C. After three PBST washes, 1 μg of PNA-HRP was introduced to each well and incubated for 2 h in the dark. The plate was then subjected to five PBST washes prior to the addition of ABTS substrate, and the absorbance at 405 nm was quantified using a plate reader. Data analysis was conducted using Microsoft Excel and GraphPad Prism 9 to determine the 50% effective enzyme concentration (EC_50_) for each NA recombinant protein.

For the assessment of neuraminidase inhibition, the 96-well plate was coated and blocked as previously detailed. Simultaneously, a distinct 96-well plate was prepared with a monoclonal antibody diluted in a 1:2 series, commencing at a concentration of 30 μg/mL, with a total of 12 dilutions, each in duplicate. The NA recombinant protein to be tested was diluted to 2 × EC_50_ and added to the wells containing the serially diluted monoclonal antibody, followed by incubation on a shaker for 1.5 h. Post-blocking of the fetuin plate with three PBST washes, the NA recombinant protein/monoclonal antibody mixture was added and incubated for 2 h at 37 °C. The experiment proceeded as described above, with data analyzed in Microsoft Excel and GraphPad Prism 9 to calculate the concentration of monoclonal antibody required to inhibit 50% of the NA protein enzymatic activity (IC_50_).

### 2.9 Bio-layer interferometry (BLI)

Bio-Layer Interferometry (BLI) was utilized to ascertain the binding affinity constants between neuraminidase (NA) recombinant proteins and the broad-spectrum antibody 1G01 (Stadlbauer et al., 2019), employing an AR2G probe for these assays. The antigen preparation involved the use of a pH 5.0 sodium acetate solution to achieve a target concentration of 30 μg/mL. Post antigen loading, the probes were blocked with a pH 8.5 ethanolamine solution to mitigate non-specific interactions. This was followed by a 5-min equilibration phase in a 0.02% PBST buffer to ensure environmental stability. The antibody was titrated against the antigen, initiating at a concentration of 100 nM with subsequent dilutions in a 3-fold gradient. The dissociation phase was executed in a 0.02% PBST buffer. Data obtained post-reaction were subjected to analysis using ForteBio Data Analysis 8.1 software and GraphPad Prism 9 to generate kinetic association and dissociation curves, providing insights into the binding kinetics of the NA recombinant proteins with 1G01.

For the quantification of antibodies within the serum of immunized mice that compete for the same epitopes as the positive control antibody 1G01, an epitope competition analysis was performed using BLI technology in conjunction with the Octet Interact analysis system. The antigen loading procedure was as previously described. A probe was first allowed to interact with 1G01 until saturation, maintained for 300 s, followed by an interaction with a mixture containing an equivalent concentration of 1G01 and the test serum for an additional 300 s. In parallel, another probe was incubated with a 0.02% PBST buffer for 300 s, prior to interaction with the test serum mixture for the same duration. Upon completion of these reactions, the data were analyzed using ForteBio Data Analysis 8.1 software to determine the competition ratio, thereby quantifying the presence of 1G01-like antibodies in the serum samples.

## 3 Results

### 3.1 N-terminal deletion mapping reveals a conserved structural boundary essential for NA solubility

**Figure 1:**
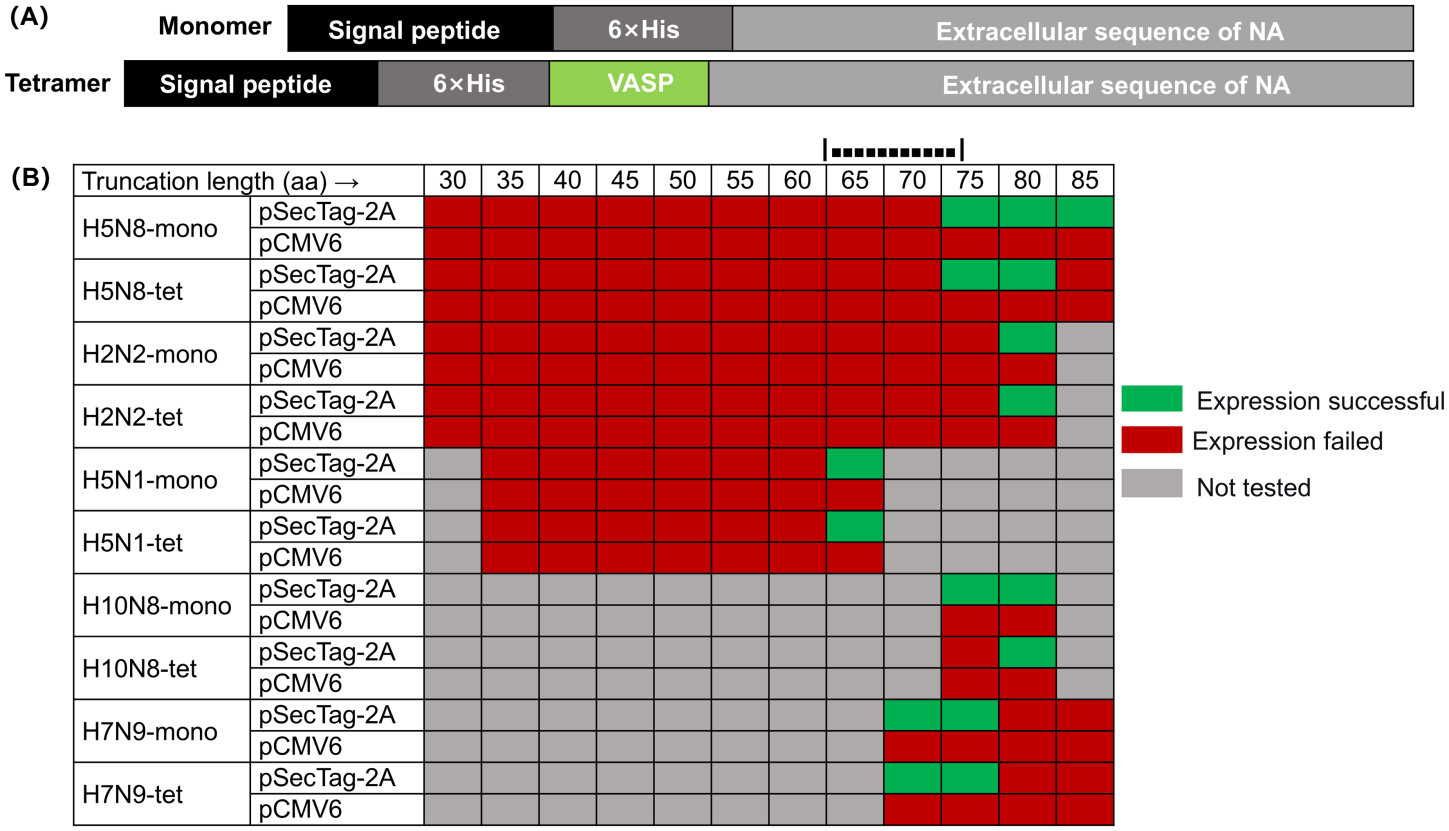
Expression profiles of NA truncation variants in different vectors. **(A)** Schematic diagram of the construction of the NA recombinant protein expression vector. The monomeric form of the NA recombinant protein is constructed by cloning the sequence encoding the extracellular domain of NA, the 6 × His tag sequence, and the DNA sequence encoding the signal peptide in the same frame; the tetrameric form of the NA recombinant protein introduces the tetrameric domain of human vasodilator-stimulated phosphoprotein (VASP) at the N-terminus. **(B)** Expression screening of NA N-terminal truncation variants in two mammalian vectors. This figure summarizes the expression results of NA proteins with varying N-terminal truncations (in both monomeric “mono” and forced tetrameric “tet” forms *via* the VASP domain) in Expi293 cells using either the pSecTag-2A or pCMV6 vector. Green indicates successful soluble expression, red indicates no soluble expression was detected, and gray indicates that the experiment was not performed for that specific combination.

NA, a type II transmembrane glycoprotein, assembles into a mushroom-shaped tetramer composed of four identical subunits, each containing a N-terminal cytoplasmic tail, a transmembrane segment, a stalk, and a globular head harboring the enzymatic active site (Nguyen-Van-Tam, 2015). As illustrated in Figure 1A, we engineered recombinant NA proteins in both monomeric and tetrameric forms, encompassing the stalk and head regions. To promote tetramer formation, a human VASP tetramerization domain was fused to the N-terminus of selected constructs, an approach previously shown to enhance structural stability and preserve enzymatic activity (Kuhnel et al., 2004; Stadlbauer et al., 2019).

To delineate the structural determinants required for soluble NA expression, we generated a series of constructs containing progressive N-terminal deletions (30–85 amino acids) and expressed them using different mammalian vectors, including pCMV6 and pSecTag-2A, in the Expi293 system. Soluble expression was consistently observed only when the N-terminal truncation ranged between approximately 65 and 80 amino acids, and exclusively with the pSecTag-2A/Expi293 platform. This pattern was reproducible across multiple subtypes (H2N2, H5N8, H5N1, H7N9, and H10N8), suggesting the presence of a conserved structural boundary within the stalk region that governs proper folding and secretion of NA (Figure 1B).

For instance, in the H5N8 subtype, constructs truncated by up to 67 residues failed to express, whereas a 79-residue deletion enabled soluble production of both monomeric and tetrameric forms. Extending the truncation to 84 residues abolished solubility, indicating that excessive removal disrupts folding. Similar expression boundaries were observed in H2N2 (80 aa), H5N1 (63 aa), H10N8 (79 aa), and H7N9 (72 aa), with the critical region consistently located within a narrow 65–80 aa window. Tetrameric constructs exhibited higher sensitivity to over-truncation, implying that residues near this region are important for inter-subunit stability.

### 3.2 Preparation and structural validation of high-purity recombinant NA proteins

After establishing the 65–80 aa truncation boundary, we next compared the expression, purification, and characterization of monomeric and tetrameric NA proteins from the H2N2 and H5N8 subtypes.

**Figure 2:**
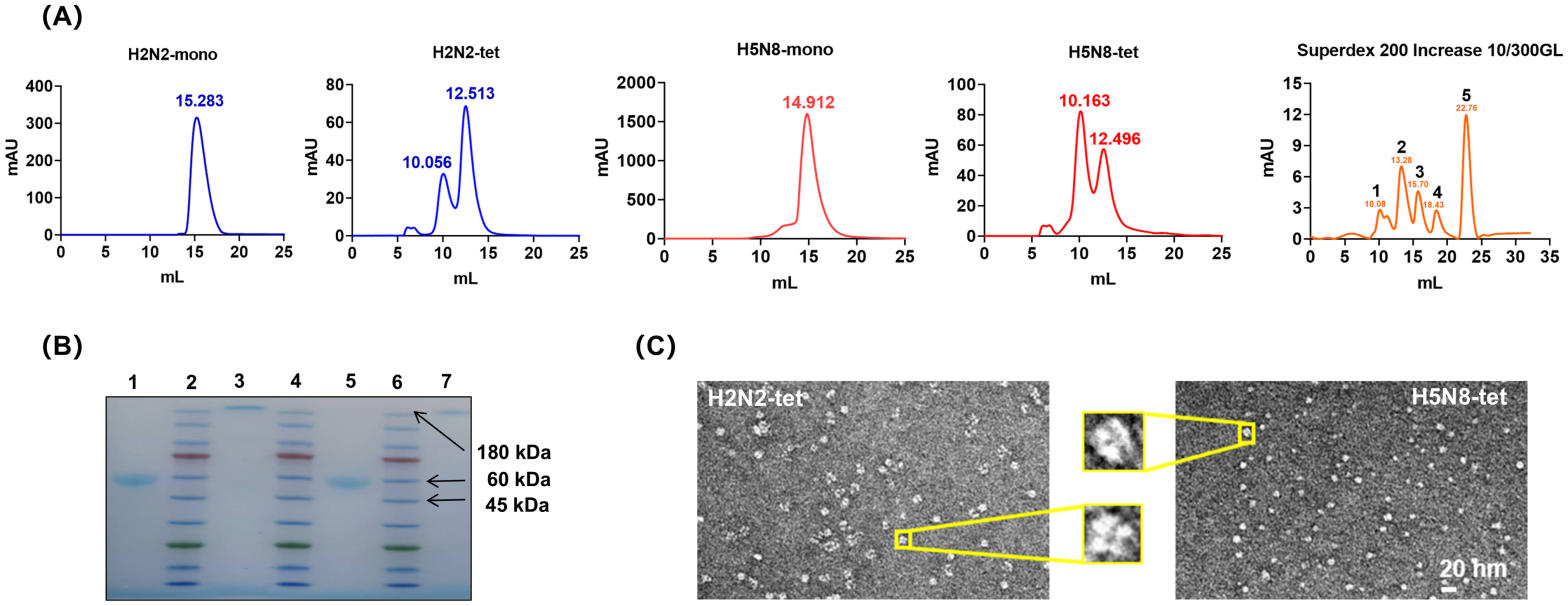
Expression and purification of NA recombinant proteins. **(A)** SEC profile of the NA recombinant proteins. The molecular weight of the monomeric NA recombinant protein is 40 ~ 45 kDa, corresponding to a SEC elution volume of approximately 15 mL; the molecular weight of the tetrameric NA recombinant protein is 160 ~ 180 kDa, corresponding to a SEC elution volume of approximately 12 mL; Superdex 200 Increase 10/300GL chromatography column markers: position 1 is 670 kDa; position 2 is 150 kDa; position 3 is 44.3 kDa; position 4 is 13.7 kDa; position 5 is 0.137 kDa. **(B)** Native-PAGE electrophoresis of the NA recombinant proteins after SEC purification. Positions 1 and 3 correspond to H5N8-mono and H5N8-tet, respectively; positions 2, 4, and 6 are protein markers; positions 5 and 7 correspond to H2N2-mono and H2N2-tet, respectively. **(C)** Negative staining electron micrographs of H2N2-tet and H5N8-tet. The image shows that both H2N2-tet and H5N8-tet proteins form typical tetrameric structures, with their four subunits symmetrically distributed, exhibiting intact morphology and compact architecture.

The culture supernatants were collected and subjected to Ni-NTA affinity chromatography followed by size exclusion chromatography (SEC) using a Superdex 200 column. The SEC profiles (Figure 2A) showed that both H2N2-mono and H5N8-mono exhibited high homogeneity, with major elution peaks at around 15 mL, corresponding to a molecular weight of approximately 45 kDa. In contrast, the elution behavior of H2N2-tet and H5N8-tet differed from that of the monomers. The tetrameric proteins tended to aggregate into high-molecular-weight multimers, with major elution peaks observed at around 12 mL, consistent with an apparent molecular weight of approximately 180 kDa.

From 1 L of culture, the yields of purified H2N2-mono and H5N8-mono were approximately 10 mg and 8 mg, respectively, while those of H2N2-tet and H5N8-tet were lower, at 5 mg and 3 mg. All purified proteins demonstrated high stability, retaining their structural integrity for over 1 month when stored at 4 °C.

As shown in Figure 2B, Native-PAGE analysis of proteins collected from the main SEC peak confirmed their purity and homogeneity, as indicated by distinct and well-defined bands. The purified samples were subsequently concentrated to 2–3 mg/mL to provide sufficient material for electron microscopy, which improved image visibility and contrast. Figure 2C presents representative negative-stain electron micrographs of H2N2-tet and H5N8-tet. Both proteins exhibited well-defined, mushroom-like tetrameric structures with four symmetrically arranged subunits. These observations confirm that fusion of the VASP tetramerization domain at the N-terminus effectively promotes proper assembly and structural symmetry of recombinant NA tetramers.

### 3.3 Enzymatic activity of NA recombinant proteins

To evaluate whether the recombinant NA proteins generated using the 65–80 amino acid truncation and forced tetramerization strategy retained their native enzymatic properties, we assessed their catalytic activity and inhibitor sensitivity using the enzyme-linked lectin assay (ELLA).

**Figure 3:**
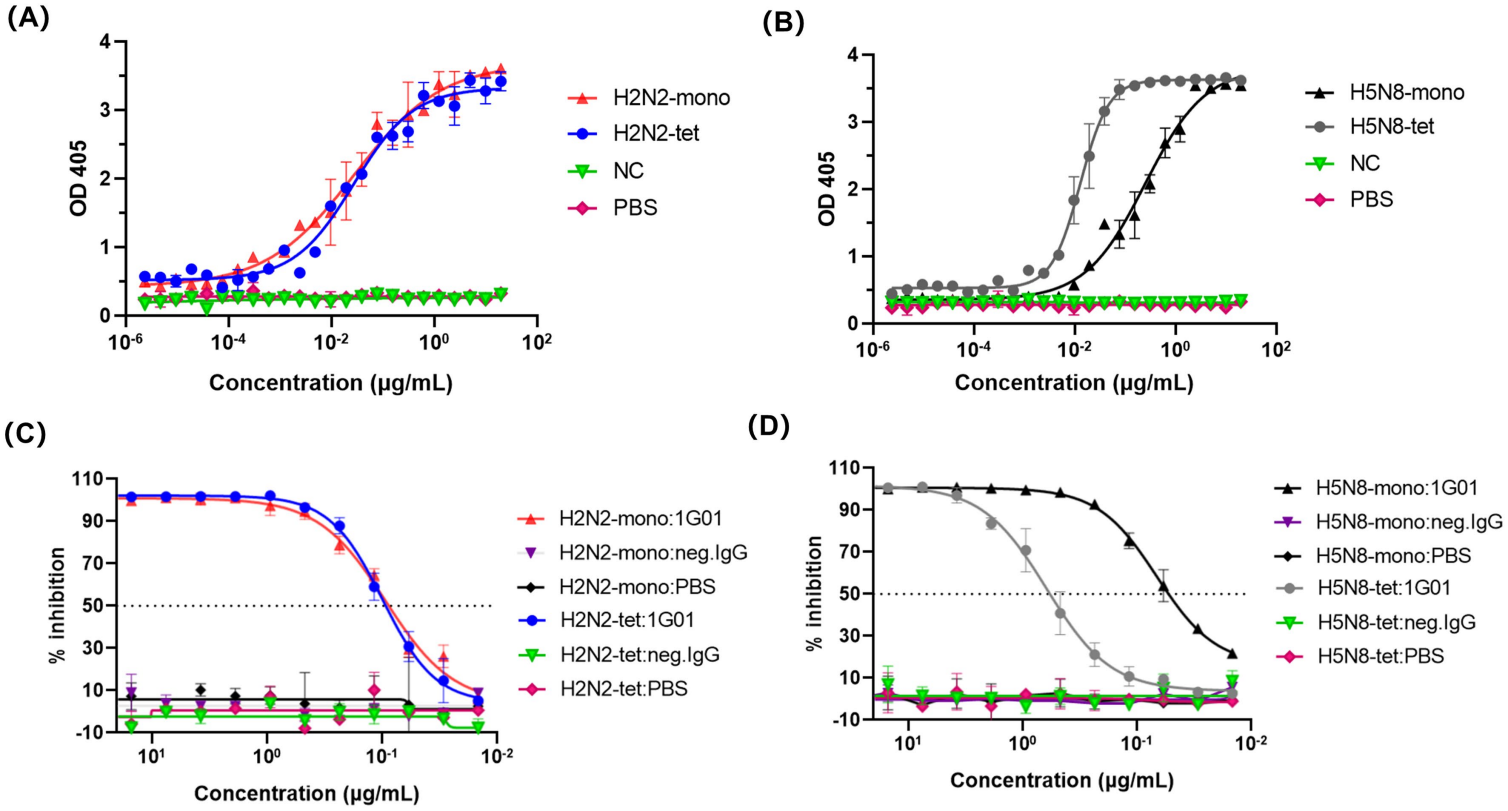
ELLA method for evaluating the enzymatic activity of NA recombinant proteins. **(A,B)** represent the determination of NA recombinant proteins enzymatic activity. In this experiment, the NA recombinant protein acts on fetuin, cutting off the terminal sialic acid residues. After the cleavage, the exposed sugar structures can be specifically recognized and bound by PNA. The enzymatic activity of the NA recombinant protein is assessed by quantifying the amount of PNA bound to fetuin. An irrelevant protein 5T4 was used as a negative control (NC), and PBS served as a blank control. **(C,D)** Depicted the inhibition of NA recombinant proteins enzymatic activity by the broadly protective NA antibody 1G01 using the ELLA method.

The enzymatic activity was assessed by quantitatively measuring the cleavage products of fetuin hydrolyzed by NA, allowing comparison of the activity between different NA protein forms. The results showed that the EC_50_ value of H2N2-tet was 0.027 μg/mL, which was not significantly different from that of its monomeric form, H2N2-mono (0.028 μg/mL) (Figure 3A). This indicates that the monomer of this subtype already adopts a fully functional catalytic conformation, and tetramerization primarily enhances structural stability without providing additional catalytic enhancement.

The EC_50_ value of H5N8-tet was 0.013 μg/mL, significantly lower than that of its monomeric counterpart, H5N8-mono (0.252 μg/mL), reflecting an approximately 20-fold increase in catalytic efficiency (Figure 3B). This enhancement likely reflects inter-subunit allosteric cooperation within the tetramer, whereas the monomeric form, lacking these stabilizing interactions, may display reduced local conformational stability and diminished catalytic activity.

We next evaluated the inhibitory effect of the broadly protective antibody 1G01 (Stadlbauer et al., 2019) on the enzymatic activity of NA. This antibody exerts its protective function by targeting the enzymatic active site of NA, thereby inhibiting the release of viral particles. As shown in Figure 3C, the IC_50_ values of 1G01 against H2N2-tet and H2N2-mono were 0.099 μg/mL and 0.093 μg/mL, respectively, indicating comparable inhibitory efficacy and further confirming that the monomeric form of this subtype maintains conformational integrity consistent with the tetramer. In contrast, the H5N8 monomer (IC_50_ = 0.067 μg/mL) was markedly more sensitive to 1G01 than its tetrameric form (IC_50_ = 0.618 μg/mL), as shown in Figure 3D. This observation aligns with the reduced enzymatic activity of H5N8-mono and suggests increased accessibility of the 1G01 epitope. The absence of inter-subunit contacts in the monomer likely alters its tertiary structure, leading to greater exposure of the active-site region and enhanced antibody-mediated inhibition.

### 3.4 Antigenicity and immunogenicity of recombinant NA proteins

**Figure 4:**
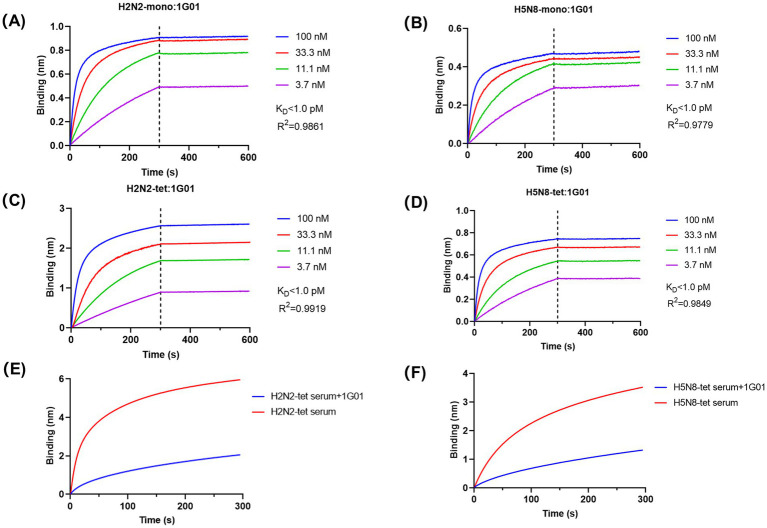
Analysis of the antigenicity and immunogenicity of NA recombinant proteins. **(A–D)** BLI technology was used to analyze the binding affinity of NA recombinant proteins with the broad-spectrum antibody 1G01. We determined the affinity constant (K_D_ value) between the NA recombinant proteins and 1G01 and calculated the curve fitting parameter R^2^ to assess the goodness of fit of the data. The calculated K_D_ values for the binding affinity of the four NA recombinant proteins with antibody 1G01 were all below 1 pM. **(E,F)** BLI technology was used to analyze the proportion of antibodies in the mouse immune serum that have the same binding epitopes as the broad-spectrum protective antibody 1G01. The red curve represents the binding curve of the antigen with antibodies in the serum, and the blue curve represents the binding curve of the antigen with 1G01 until saturation is reached, followed by the binding curve with a mixture of serum and 1G01. The proportion of antibodies in the H2N2-tet immune serum that compete for epitopes with 1G01 was calculated to be 65.91%; the proportion of antibodies in the H5N8-tet immune serum that compete for epitopes with 1G01 was calculated to be 62.74%.

To determine whether the antigenicity of the recombinant NA proteins was preserved, we measured the binding affinity between the broadly neutralizing antibody 1G01 and the NA proteins using BLI. The results demonstrated that 1G01 efficiently recognized all NA recombinant proteins with exceptionally high binding affinity, exhibiting equilibrium dissociation constant (K_D_) values below 1 pM for all constructs (Figures 4A–D). Although enzymatic activities varied among the different NA proteins, their binding affinity to 1G01 remained highly consistent, indicating that the recombinant proteins retained native antigenic characteristics. These results confirm that the 65–80 aa truncation removed only non-essential structural regions without compromising the integrity of the enzymatic active site or key epitopes. Furthermore, VASP-mediated tetramerization did not introduce significant steric hindrance.

To evaluate the immunogenicity of the recombinant NA proteins, mice were immunized with the tetrameric forms H2N2-tet and H5N8-tet, respectively, and the serum antibody binding to the corresponding antigens was analyzed (Figures 4E,F). Both proteins effectively induced immune responses and generated polyclonal antibodies. To further characterize the quality of the antibody response, competition assays were performed to determine the proportion of antibodies in the immune serum that target the same epitope as 1G01. The results showed that 65.91% of antibodies in the H2N2-tet immunized serum and 62.74% in the H5N8-tet group shared the epitope with 1G01, indicating that the majority of induced antibodies specifically recognized the conserved epitope targeted by 1G01. These data suggest that the induced antibodies have the potential to inhibit viral release through NA blockade.

In summary, the tetrameric NA recombinant proteins not only elicited strong immune responses but also directed antibody production toward conserved, functionally protective epitopes.

## 4 Discussion

Efficient production of soluble and functionally NA has long been a technical bottleneck in influenza research (Ellis et al., 2022; McMahon et al., 2020). NA is inherently membrane-associated, and recombinant expression often yields insoluble or misfolded products, particularly when attempting to preserve the native tetrameric configuration (da Silva et al., 2013; McAuley et al., 2019). In this study, we systematically optimized NA expression by combining an N-terminal truncation with enforced tetramerization (Xu et al., 2008; Zhu et al., 2019). Our results demonstrate that removal of approximately 65–80 amino acids from the N-terminus, together with expression in the pSecTag-2A/Expi293 system, enables soluble production of NA across phylogenetically diverse influenza A subtypes. Fusion of a human VASP tetramerization domain further promoted assembly into a symmetric, mushroom-like tetrameric architecture with high enzymatic activity and preserved antigenicity. These findings collectively provide a rational framework for reconciling the long-standing trade-off between solubility, structural fidelity, and functional integrity in recombinant NA production.

A key outcome of this work is the identification of a conserved N-terminal structural boundary that determines whether NA can fold and assemble properly. Previous studies typically relied on empirical truncation of individual subtypes, often yielding inconsistent results and limited scalability. By systematically scanning multiple NA subtypes, we uncovered a common truncation window (65–80 residues) that delineates the transition between the hydrophobic transmembrane-stalk region and the more soluble head domain. This region likely represents a folding-sensitive boundary where inappropriate exposure of hydrophobic residues triggers protein aggregation or misfolding during secretion (da Silva et al., 2013). Moderate truncation within this boundary removes the aggregation-prone segment while retaining key residues essential for inter-subunit interactions near the neck domain, thereby promoting correct quaternary assembly. The addition of the VASP tetramerization motif provides further stabilization, guiding the formation of the native mushroom-like tetrameric architecture observed under electron microscopy. The sharp solubility loss observed when truncation exceeded ~80 residues underscores the structural precision of this boundary and highlights the balance required between eliminating hydrophobic sequences and preserving oligomerization interfaces.

The use of the Expi293 mammalian system further contributed to the functional integrity of the recombinant proteins. Unlike insect or bacterial systems, Expi293 cells provide complex-type glycosylation, which prevents high-mannose modifications that have been reported to compromise NA enzymatic activity and antibody recognition (van der Woude et al., 2020). The high-affinity binding of our recombinant NAs to the broadly protective antibody 1G01 confirms that this strategy preserves the native antigenic conformation, including the conserved catalytic-site epitope. Moreover, the marked enhancement of catalytic efficiency in the tetrameric form of H5N8 (approximately 20-fold over the monomer, Figure 3B) indicates an allosteric effect mediated by inter-subunit interactions. Importantly, immunization with tetrameric NAs elicited antibodies that predominantly targeted the same conserved epitope as 1G01 (Figures 4E,F), suggesting that the engineered tetramers not only maintain structural authenticity but also bias the immune response toward functionally protective epitopes.

Collectively, these findings provide mechanistic insight into the structural requirements for soluble and immunogenic NA expression. The defined N-terminal boundary and tetramerization-based stabilization offer a broadly applicable design principle for recombinant NA production across influenza A subtypes. Based on the comprehensive antigenic epitope profile presented by this platform, systematic discovery of biologics targeting conserved epitopes of influenza viruses can be achieved. The application scope extends from traditional neutralizing antibodies to non-neutralizing antibodies that function through effector mechanisms such as ADCC, thereby providing diverse candidate molecules for enriched influenza prevention and control strategies (Wang et al., 2020; Wu et al., 2017; Yu et al., 2017). Beyond facilitating structural and biochemical studies, this approach may accelerate the development of NA-based universal influenza vaccines and improve the screening of next-generation NA inhibitors (Abbadi and Mousa, 2023). More broadly, the concept of combining rational truncation with controlled oligomerization may be extended to other viral or membrane-associated glycoproteins that suffer from similar solubility-function trade-offs (Wu and Ellebedy, 2024).

## Data Availability

The raw data supporting the conclusions of this article will be made available by the authors, without undue reservation.
